# Changes in BMI and physical activity from youth to adulthood distinguish normal-weight, metabolically obese adults from those who remain healthy

**DOI:** 10.3389/fendo.2022.923327

**Published:** 2022-08-10

**Authors:** A. Viitasalo, K. Pahkala, T. Lehtimäki, JSA. Viikari, TH. Tammelin, O. Raitakari, TO. Kilpeläinen

**Affiliations:** ^1^ Institute of Biomedicine, School of Medicine, University of Eastern Finland, Kuopio, Finland; ^2^ Centre for Population Health Research, University of Turku and Turku University Hospital, Turku, Finland; ^3^ Research Centre of Applied and Preventive Cardiovascular Medicine, University of Turku, Turku, Finland; ^4^ Paavo Nurmi Centre & Unit for Health and Physical Activity, University of Turku, Turku, Finland; ^5^ Department of Clinical Chemistry, Fimlab Laboratories, Tampere, Finland; ^6^ Faculty of Medicine and Health Technology, Finnish Cardiovascular Research Center, Tampere University, Tampere, Finland; ^7^ Department of Medicine, University of Turku and Division of Medicine, Turku University Hospital, Turku, Finland; ^8^ JAMK University of Applied Sciences, LIKES, Jyväskylä, Finland; ^9^ Department of Clinical Physiology and Nuclear Medicine, Turku University Hospital, Turku, Finland; ^10^ Novo Nordisk Foundation Center for Basic Metabolic Research, Faculty of Health and Medical Sciences, University of Copenhagen, Copenhagen, Denmark

**Keywords:** child, normal weight, metabolic risk, obesity, physical activity, BMI, adult, follow-up

## Abstract

**Highlights:**

Adults with MONW have a lower BMI during youth until young adulthood, but higher BMI after this than adults with metabolically healthy normal weight. Adults with MONW have a greater decrease in physical activity from youth to adulthood than other adults. Healthy lifestyle is important in the prevention of metabolic disorders, particularly in individuals who are slim in childhood.

**Background:**

Individuals with metabolically obese normal-weight (MONW) have higher risk of cardiovascular events than those with obesity but a metabolically healthy status. Etiological factors leading to MONW are not well known. We hypothesized distinct trajectories of changes in BMI and physical activity may modify metabolic risk and distinguish individuals with MONW from those who remain healthy.

**Methods:**

We compared the mean levels of BMI and physical activity at eight time points (1980, 1983, 1986, 1989, 1992, 2001, 2007, 2011) between MONW and healthy normal-weight adults using linear mixed-model analysis. The analyses included 1180 participants of the Cardiovascular Risk in Young Finns study, a population-based study that represents six different age cohorts 3, 6, 9, 12, 15 and 18 years of age at baseline.

**Results:**

Individuals with adult MONW had significantly lower BMI in childhood and young adulthood, but their BMI increased more than in other adults after this age (p<0.001for interaction between time and MONW status). Physical activity decreased relatively more since youth in individuals with adult MONW (p<0.001).

**Conclusions:**

Relative leanness in youth and subsequent weight gain in young adulthood, and a gradual decrease in physical activity levels from youth to adulthood, predispose normal-weight individuals to metabolic impairments. The results highlight the importance of a healthy lifestyle in the prevention of metabolic disorders, particularly in individuals who are slim in childhood.

## Introduction

Overweight and obesity are associated with an adverse metabolic risk profile and increased risk of type 2 diabetes and cardiovascular disease. However, some adults with normal weight show a metabolic risk profile similar to individuals with obesity, despite being lean (1). This condition, “metabolically obese normal-weight” (MONW), has been found to be associated with higher risk of cardiovascular events than that in adults with obesity but a metabolically healthy status (2). Nevertheless, individuals with MONW are often ignored in screening and prevention efforts, and the etiological factors leading to MONW remain poorly understood.

Studies of adult obesity (3–5) indicate that at the same level of adult BMI, individuals who gain most weight from childhood to adulthood have the highest cardiometabolic risk. We have recently shown that, even among individuals who retain normal-weight in adulthood, a relatively higher weight gain from childhood to adulthood is associated with a MONW profile (6). However, it is currently unclear whether the association between weight gain and MONW is characterized by a distinct trajectory of changes in BMI, in which weight gain occurs at a specific critical time point during childhood, adolescence or adulthood (7–9). Furthermore, while previous studies suggest that adults with MONW are less physically active than other adults (10), it remains unclear whether distinct trajectories of changes in physical activity could modify metabolic risk and distinguish individuals with MONW from those who remain healthy (10, 11).

Longitudinal follow-up is critical for understanding the influence of youth-to-adulthood changes in BMI and physical activity on metabolic health in individuals with normal weight. In the present study, we examine the association of youth-to-adulthood BMI and physical activity changes from age 3 to 49 years with MONW status in the Cardiovascular Risk in Young Finns study (YFS).

## Methods

### Study design and measurements

The Cardiovascular Risk in Young Finns Study is an ongoing population-based follow-up study of atherosclerotic precursors (12). In 1980, a total of 4 320 Finnish children representing six different age cohorts (3, 6, 9, 12, 15, and 18 years of age) were invited, and 3 596 (83.2%) children participated in the first cross-sectional survey. The follow-up studies were performed in 1983, 1986, 1989, 1992, 2001, 2007 and 2011 (flow chart, **Supplementary Figure S1**). The study was approved by the Ethics Committee of Hospital District of Southwest Finland in agreement with the Declaration of Helsinki, and all participants provided written informed consent. Adults with underweight (BMI<18.5 kg/m^2^), overweight (BMI>25 kg/m^2^), type 1 diabetes or pregnancy in adulthood were excluded from the present analyses.

Height and weight were measured, and BMI was calculated as weight in kilograms divided by height in meters squared. Physical activity was measured with a standardized self-administered questionnaire in all study phases from the age of nine in/beginning from 1980 (13). The self-administered questionnaire included questions concerning the frequency and intensity of leisure-time physical activity, participation in sports club training, participation in competitive sport events, and the habitual way of spending leisure time. A physical activity index was calculated as previously described (range 5–15) (14). Validation of the Young Finns Study physical activity data has been done previously (15). Socioeconomic position was assessed in 2001 by occupational status (manual; lower-grade non-manual; and higher-grade non-manual) (16). Ultrasound imaging of the liver was performed using a validated protocol https://www.ncbi.nlm.nih.gov/pmc/articles/ PMC6037671/- CR17 and Sequoia 512 ultrasound mainframes (Acuson, Mountain View, CA, USA) with 4.0 MHz adult abdominal transducers in 2011. Evaluation of hepatic steatosis was performed according to liver-to-kidney contrast, parenchymal brightness, deep beam attenuation, and bright vessel walls. According to these criteria the presence of hepatic steatosis was assessed visually from images by a highly trained ultrasonographer.

Blood pressure was measured from the brachial artery with a standard mercury sphygmomanometer in childhood and with a random zero sphygmomanometer in adulthood. The average of three measurements was used in the statistical analyses. Venous blood samples were drawn after an overnight fast for determination of lipid and serum glucose concentrations. Standard enzymatic methods were used for serum triglycerides and high-density lipoprotein cholesterol (12, 17).

### Definition of the metabolically obese normal-weight phenotype

MONW was defined as BMI 18.5-25.0 kg/m^2^ in the presence of two or more components of the International Diabetes Federation (IDF) criteria for the metabolic syndrome (hypertriglyceridemia, low HDL cholesterol, high blood pressure, high fasting glucose) (18). All other normal-weight individuals (BMI 18.5-25.0 kg/m^2^) were defined as metabolically healthy normal-weight. The cut-off points for the risk factors were as follows: hypertriglyceridemia: ≥ 1.7 mmol/L; low HDL cholesterol: < 1.03 mmol/L in males and < 1.29 mmol/L in females, or treatment for hypercholesterolemia; high blood pressure: systolic blood pressure ≥ 130 or diastolic blood pressure ≥ 85 mm Hg, or treatment of previously diagnosed hypertension; high fasting glucose: ≥ 5.6 mmol/L, or previously diagnosed type 2 diabetes. Participants were classified as MONW or metabolically healthy at the latest adulthood follow-up (2011, 2007 or 2001) where they had the data required to define metabolic health and BMI.

### Statistical analysis

We compared the mean levels of BMI and physical activity from youth to adulthood between those with MONW and those who were metabolically healthy using linear mixed-model analysis, by entering MONW status, age (time, modelled as a categorical variable), length of follow-up (to account for different follow-up times), sex and MONW status × age interaction terms as fixed covariates and participant as a random effect in the model. The models were additionally adjusted for socioeconomic status, and physical activity or BMI. We also performed additional analyses stratified by sex. Mixed models use all available data, assuming missing data is missing at random. Differences and interactions with the p-values of <0.05 were considered statistically significant. Statistical analyses were performed with the IBM SPSS Statistics software, Version 21 (IBM Corp., Armonk, NY).

## Results

Characteristics of the study participants at baseline and at the end of follow up are shown in **Table 1**. Participants who developed MONW in adulthood had modestly higher triglycerides, and systolic and diastolic blood pressure, and lower HDL cholesterol than their metabolically healthy normal weight peers already at baseline at the age of 3-18 years. The participants who developed MONW were also more often males (57.9 vs 35.7%) and older (mean 11.6 (range 2.9-18.9) vs. 10.2 (2.7-18.9) years at baseline; 37.7 (24.0-49.8) vs. 36.2 (23.8-50.2) years in average at adulthood follow-ups). After adjusting for sex and age, the differences in baseline systolic and diastolic blood pressure were no longer significant. After these adjustments, baseline BMI was lower in participants who developed MONW (mean 16.5 (range 16.9-17.1) kg/m^2^) compared to those who were metabolically healthy normal weight adults (17.0 (16.9-17.1) kg/m^2^). On the other hand, adult BMI was higher in participants who developed MONW than in participants with healthy normal weight.

**Table 1:** Characteristics of participants with metabolically healthy or metabolically obese normal weight status in adulthood.

	Metabolically healthy normal weight (n=1002)	Metabolically obese normal weight (n=178)	p-value	p-value adj. forage and sex
Male sex (%)	35.7	57.9	**3.8x10^-8^**	
**Baseline (1980)**				
Age (years)	10.2 (2.7-18.9)	11.6 (2.9-18.9)	**4.2x10^-4^**	
BMI (kg/m^2^)	16.9 (2.6)	16.9 (2.3)	0.880	**0.001**
Triglycerides (mmol/L)	0.64 (0.29)	0.71 (0.30)	**0.004**	**0.006**
HDL cholesterol (mmol/L)	1.6 (0.3)	1.5 (0.3)	**1.0x10^-6^**	**2.0x10^-6^**
Systolic blood pressure (mmHg)	110 (12)	114 (13)	**0.001**	0.051
Diastolic blood pressure (mmHg)	68 (9)	70 (10)	**0.015**	0.085
Physical activity index*	9.0 (1.7)	9.0 (1.9)	0.654	0.782
**Adulthood (2001,2007,2011)**				
Age (years)	36.2 (23.8-50.2)	37.7 (24.0-49.8)	**6.0x10^-6^**	
BMI (kg/m^2^)	22.2 (1.9)	22.9 (2.0)	**1.6x10^-10^**	**1.5x10^-4^**
Triglycerides (mmol/L)	1.00 (0.46)	1.59 (1.89)	**6.4x10^-10^**	**7.8x10^-36^**
HDL cholesterol (mmol/L)	1.4 (0.3)	1.2 (0.3)	**1.5x10^-44^**	**2.0x10^-36^**
Systolic blood pressure (mmHg)	113 (12)	125 (15)	**1.1x10^-41^**	**4.3x10^-36^**
Diastolic blood pressure (mmHg)	69 (9)	77 (11)	**4.5x10^-36^**	**5.3x10^-34^**
Physical activity index	9.1 (1.9)	8.6 (1.7)	**5.5x10^-47^**	**5.6x10^-5^**
Prevalence of fatty liver (2011) (%)	4.3	13.8	**5.7x10^-13^**	**1.0x10^-4^**
Socioeconomic status (2001) (manual; lower-grade non-manual; and higher-grade non-manual) (%)	28/44/28	39/41/20	**6.0x10^-6^**	

Values are means (standard deviations) or means (range) for continuous variables and percentages for categorical variables. P -values are from Independent Samples T-test and Chi-squared test.

Information on number of participants with data on BMI and physical activity index at each follow-up are presented in Flow chart, **Supplementary Figure S1**. P-values <0.05 are bolded.

### Changes in BMI from youth to adulthood

**Figure 1:**
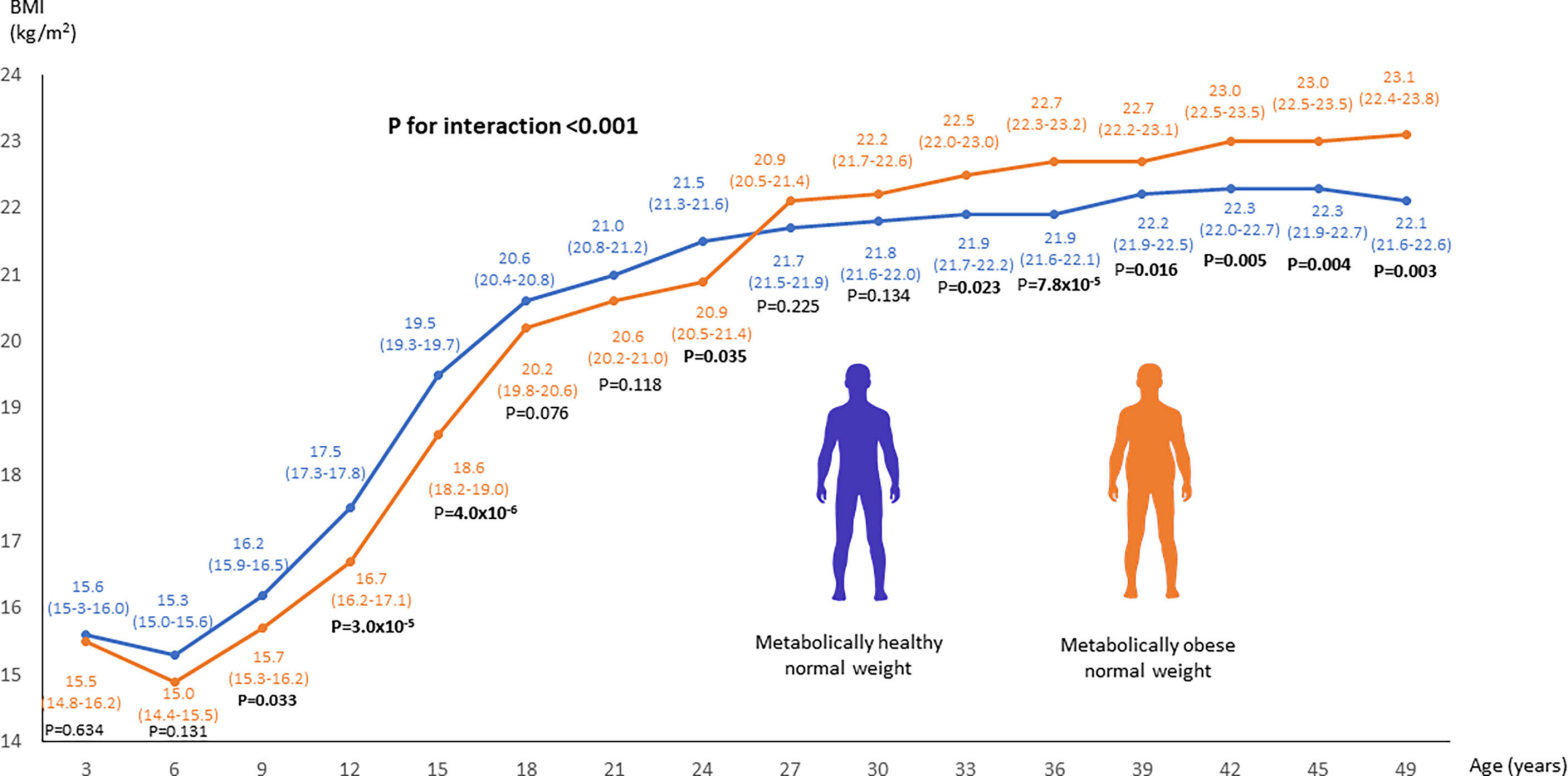
Linear mixed model analysis to compare the changes in BMI from youth to adulthood between adults with MONW and metabolically healthy normal weight. MONW phenotype was defined as the presence of two or more components of the metabolic syndrome according to the IDF criteria. P-values <0.05 are bolded.

Individuals with adult MONW had significantly lower BMI in childhood and young adulthood, from 9 years to 24 years of age, but their BMI increased relatively more than in other adults after this age and was significantly higher from 33 years onwards. The interaction between time and MONW status was significant (p<0.001), indicating that the BMI trajectory over time modified MONW risk (**Figure 1**). Additional adjustment for physical activity levels at each measurement time point (p<0.001) or socioeconomic status (**Supplementary Figure S2**) in adulthood (p<0.001) did not have a major effect on the results. In the sex-stratified analyses, the changes of BMI in participants with MONW and metabolically healthy normal weight were similar among men (p<0.001) and women (p<0.001) (**Supplementary Figures S3, S4**).

### Changes in physical activity from youth to adulthood

**Figure 2:**
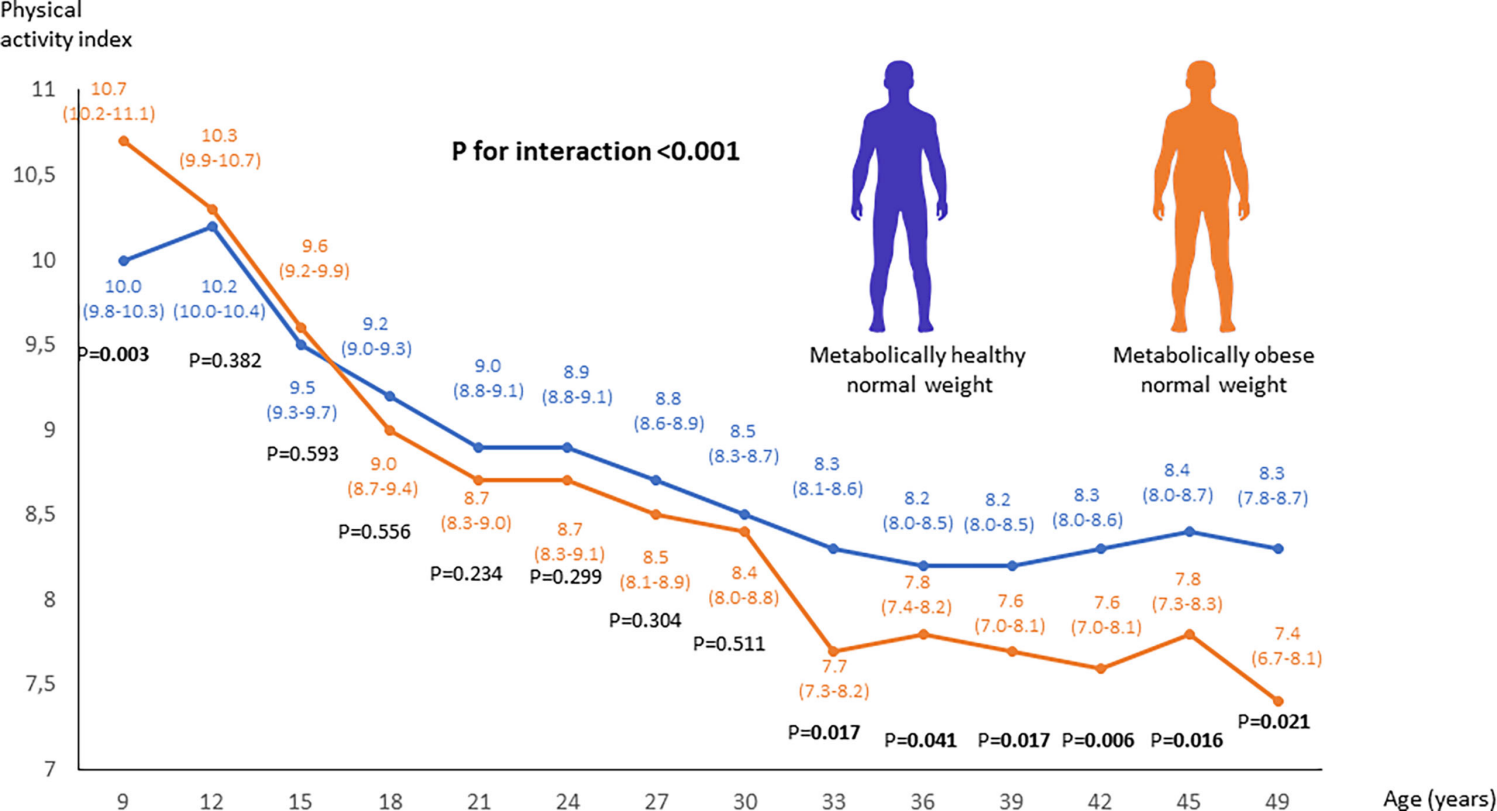
Linear mixed model analysis to compare the changes in physical activity from youth to adulthood between adults with MONW and metabolically healthy normal weight. MONW phenotype was defined as the presence of two or more components of the metabolic syndrome according to the IDF criteria.

Individuals with adult MONW were physically more active at 9 years of age than other individuals, but their physical activity decreased relatively more after this age, reaching significantly lower levels from 33 years of age onwards. A significant interaction between follow-up time and MONW status was observed (p<0.001), indicating that the trajectory of physical activity over time modified MONW risk (**Figure 2**). The results remained statistically significant after additional adjustment for BMI at each measurement time point (p=0.001) and after adjustment for socioeconomic status (**Supplementary Figure S5**) in adulthood (p=0.015). When stratifying the analyses according to sex, the difference in changes of physical activity over time between participants with MONW and metabolically healthy normal weight were more pronounced in men (p=0.004) than in women (p=0.783) (**Supplementary Figures S6, S7**).

## Discussion

We found that adults with MONW have a lower BMI during youth until young adulthood (age 24),

but show a higher BMI after this (from age 27 onwards) than adults with metabolically healthy normal weight. We also found that adults with MONW have a greater decrease in physical activity from youth to adulthood than other adults. Taken together, the results suggest that relative leanness in youth and subsequent weight gain in young adulthood, and a gradual decrease in physical activity levels from youth to adulthood, predispose metabolic impairments for normal-weight individuals.

The present study is, to our knowledge, the first to compare the changes in BMI and physical activity from youth to adulthood between normal-weight adults with a metabolically obese or healthy status. A recent study from the Young Finns Study suggested that a metabolically healthy profile in adults with obesity is characterized by a lower adult BMI, but not youth BMI (3). Other studies have supported the view that the accuracy for childhood BMI to predict adult morbidity is low, because the majority of obesity-related morbidity occurs in adults who had normal weight in childhood, and because most children in the population have normal-weight (19). Furthermore, in previous studies lower childhood BMI with early adiposity rebound has been associated with higher cardiometabolic risk in obese adults (4, 5). In these studies, the greatest disease risk occurred in individuals who gained the most weight from childhood to adulthood. The present results suggest that the relationship between lower BMI in childhood and higher cardiometabolic risk in adulthood is also true for individuals who retain normal weight in adulthood. It is possible that adipose tissue depots do not adapt to store high amounts of fat during childhood in individuals with low childhood BMI. Radiocarbon dating experiments have shown that the total number of adipocytes is determined in childhood and remains virtually unchanged in adulthood (20). Leanness in childhood may limit the number of adipocytes that are available for storing fat. If weight gain later occurs, the low number of adipocytes will lead to excess adipocyte hypertrophy, a key determinant in the development of obesity-related insulin resistance and related comorbidities (21). Therefore, individuals who are lean in childhood may exceed their fat storage capacity in a relatively small weight window and develop metabolic abnormalities (22).

In our data, the age from 24 to 27 years represented a cross-over point where lean individuals who developed adult MONW, surpassed the BMI of individuals who remained metabolically healthy. This age was also characterized by a relatively high weight gain in MONW adults, compared to those who remained healthy. Young adulthood often involves major changes in lifestyle due to changing responsibilities, such as full-time work and starting a family, which may contribute to weight gain. The individuals who became MONW had lower socioeconomic status than those who remained metabolically healthy. This could drive changes in lifestyle that drive increased weight gain, particularly in young adulthood.

Previous studies suggest that adults with MONW are less physically active than other adults (10). In conjunction with the previous studies, we found that physical activity levels were lower in adults with MONW compared to metabolically healthy adults with normal weight (10). However, we also found that this difference was not apparent in childhood. Rather, there was a gradual decrease in physical activity levels from youth to adulthood in individuals who developed MONW. The influence of the physical activity changes on MONW risk was independent of changes in BMI. Our findings suggest that maintaining higher physical activity levels over the life course is important in the prevention of metabolic disorders in individuals who retain normal-weight, independent of changes in body weight. Similar to physical activity, dietary factors could play an important role in metabolic risk in normal-weight individuals. Further studies are needed to investigate the role of dietary factors from childhood to adulthood in the development of MONW.

Although our results were independent of occupational status of the participants, complicated patterns related to socioeconomic status could play a role in the development of MONW. Besides socioeconomic factors, genetic factors affect metabolic risk. In our study, adults with MONW had higher triglyceride and lower HDL levels already in childhood, which could reflect the effect of both early environmental factors and the effect of genetic risk factors.

We found that the gradual decrease in physical activity and higher increase in BMI observed in the MONW group, compared to those who remained metabolically healthy, continued until the very end of the survey period when the participants reached the age 49. There were no differences between the MONW and healthy normal-weight groups in the length of the follow-up period, which suggests that the differences were independent of the follow-up time. Nevertheless, future studies are needed to examine whether the same trends in physical activity and BMI continue also in older age, and when the MONW status in defined at older age.

In conclusion, we found that MONW is characterized by a leaner childhood phenotype but relatively higher weight gain in young adulthood, and a greater decrease in physical activity levels from childhood to adulthood. Our findings indicate that health recommendations to avoid unhealthy weight gain and to be physically active are important also for individuals who retain normal weight, to prevent cardiovascular and metabolic disorders.

## Data availability statement

The datasets presented in this article are not readily available because of confidentiality issues. Requests to access the datasets should be directed to anna.viitasalo@uef.fi.

## Ethics statement

The studies involving human participants were reviewed and approved by Ethics Committee of Hospital District of Southwest Finland in agreement with the Declaration of Helsinki. Written informed consent to participate in this study was provided by the participants’ legal guardian/next of kin.

## Author contribution

AV researched data. AV and TK designed research and wrote the manuscript. Other co-authors conducted research and/or provided essential materials. AV had primary responsibility for the final content. All authors read and approved the final manuscript.

## Funding

This project was supported by North Savonia Regional Fund of Finnish Cultural Foundation, Juho Vainio Foundation and The Diabetes Research Foundation of Finland. Tuomas O. Kilpeläinen was funded by the Novo Nordisk Foundation (grants NNF18CC0034900 and NNF20OC0063707). The Young Finns Study has been financially supported by the Academy of Finland: grants 322098, 286284, 134309 (Eye), 126925, 121584, 124282, 129378 (Salve), 117787 (Gendi), and 41071 (Skidi); the Social Insurance Institution of Finland; Competitive State Research Financing of the Expert Responsibility area of Kuopio, Tampere and Turku University Hospitals (grant X51001); Juho Vainio Foundation; Paavo Nurmi Foundation; Finnish Foundation for Cardiovascular Research; Finnish Cultural Foundation; The Sigrid Juselius Foundation; Tampere Tuberculosis Foundation; Emil Aaltonen Foundation; Yrjö Jahnsson Foundation; Signe and Ane Gyllenberg Foundation; Diabetes Research Foundation of Finnish Diabetes Association; This project has received funding from the European Union’s Horizon 2020 research and innovation programme under grant agreements No 848146 for To Aition and grant agreement 755320 for TAXINOMISIS; European Research Council (grant 742927 for MULTIEPIGEN project); Tampere University Hospital Supporting Foundation and Finnish Society of Clinical Chemistry.

## Acknowledgments

We thank Oscar Lopez Astaiza for assistance with creating the human silhouette icons (**Figures 1**, **2**) (contact: oscar.lopez.astaiza@gmail.com).

## Conflict of interest

TL was an employee of Fimlab Laboratories.

The remaining authors declare that the research was conducted in the absence of any commercial or financial relationships that could be construed as a potential conflict of interest.

## Publisher’s note

All claims expressed in this article are solely those of the authors and do not necessarily represent those of their affiliated organizations, or those of the publisher, the editors and the reviewers. Any product that may be evaluated in this article, or claim that may be made by its manufacturer, is not guaranteed or endorsed by the publisher.
