# Supplementary figures and images for: Changes in BMI and physical activity from youth to adulthood distinguish normal-weight, metabolically obese adults from those who remain healthy
Source: Front Endocrinol (Lausanne). 2022 Aug 10;13:923327. doi: 10.3389/fendo.2022.923327 (PMC9399835; doi:10.3389/fendo.2022.923327)

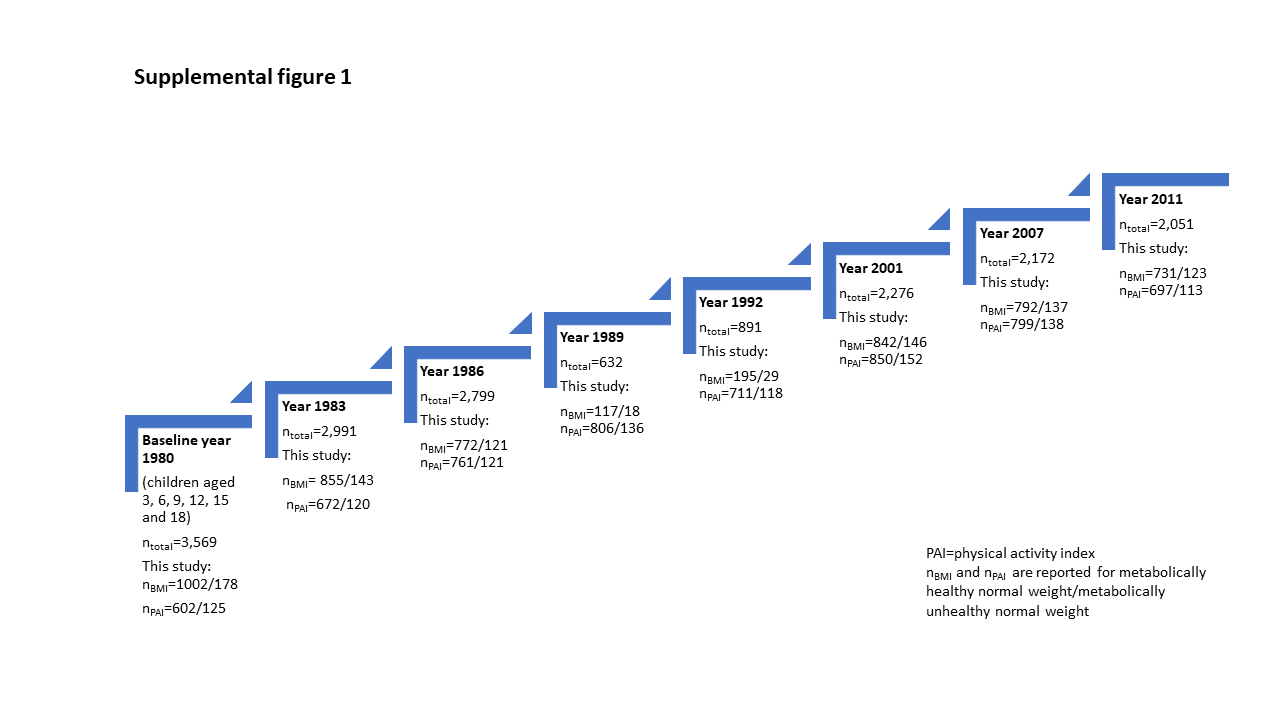

Supplement: Supplementary file 1 [file Image_1.tif]

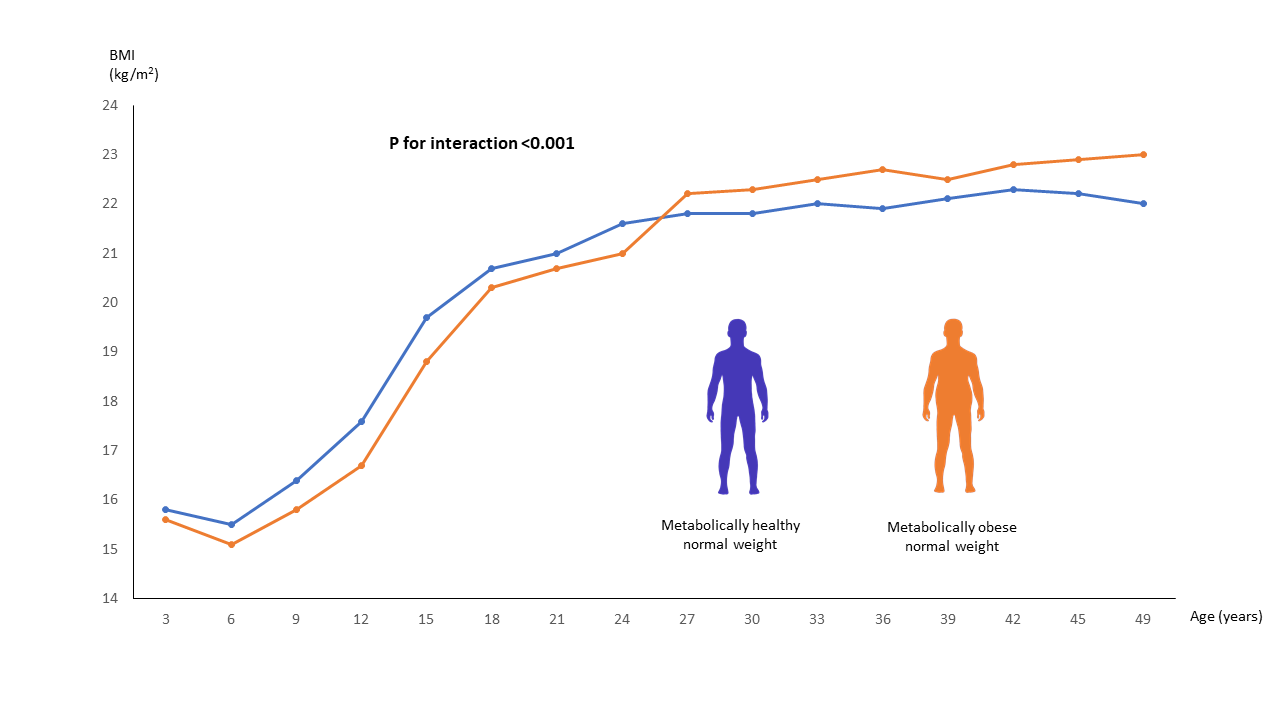

Supplement: Supplementary file 2 [file Image_2.tif]

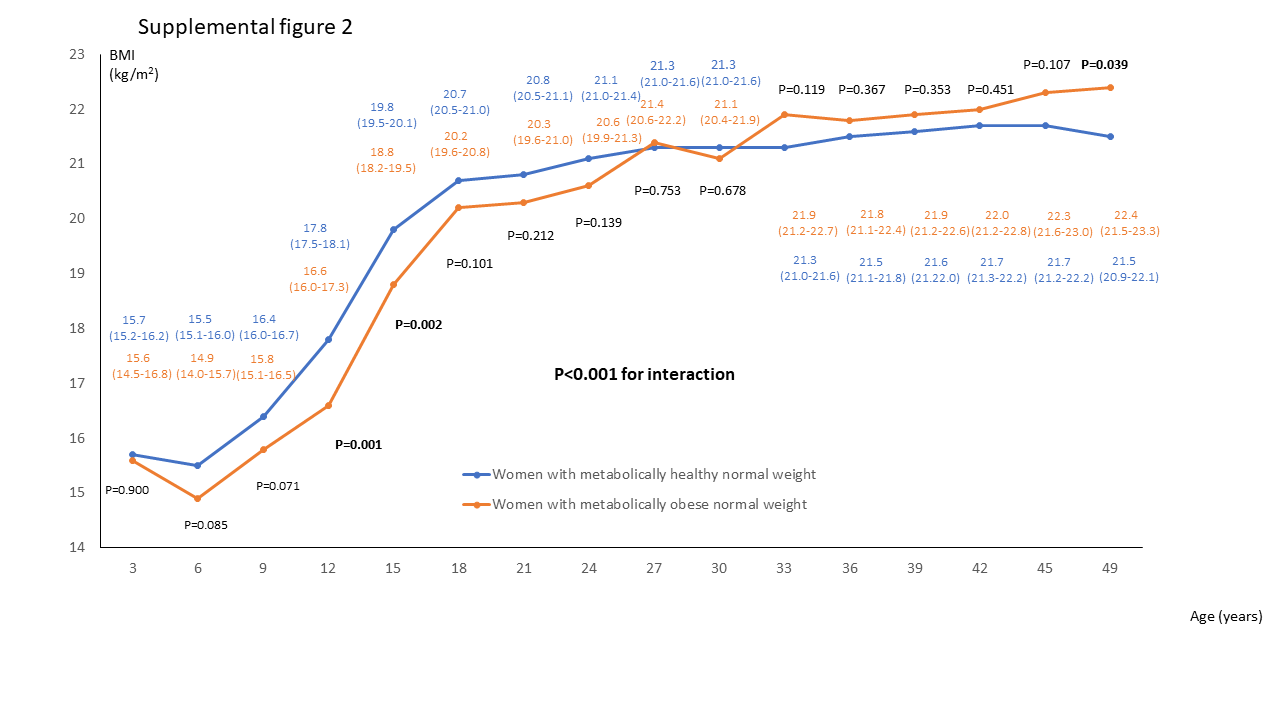

Supplement: Supplementary file 3 [file Image_3.tif]

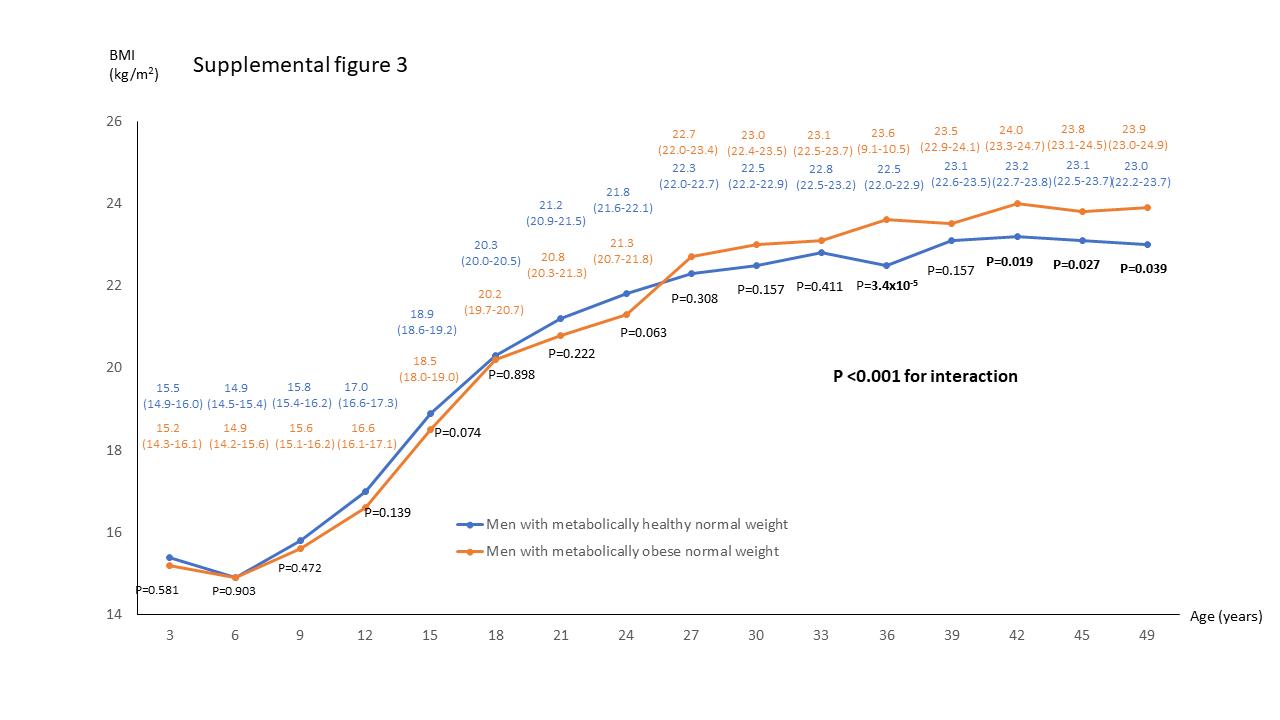

Supplement: Supplementary file 4 [file Image_4.tif]

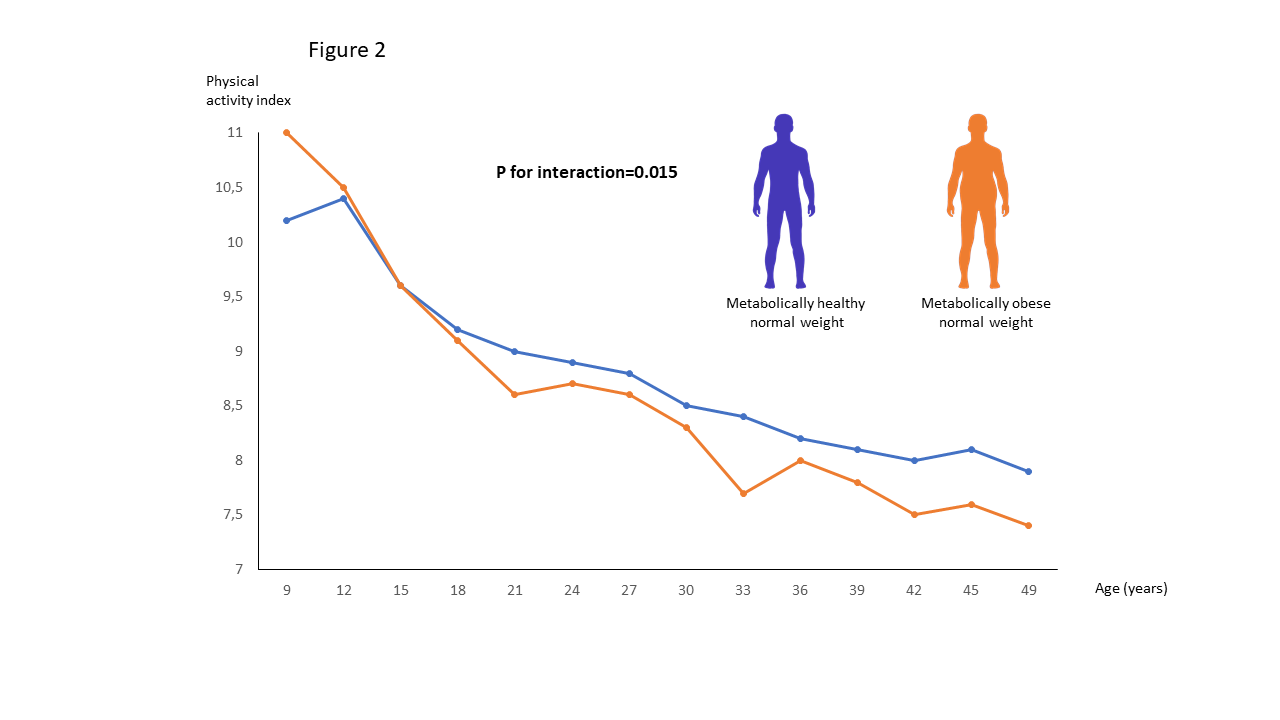

Supplement: Supplementary file 5 [file Image_5.tif]

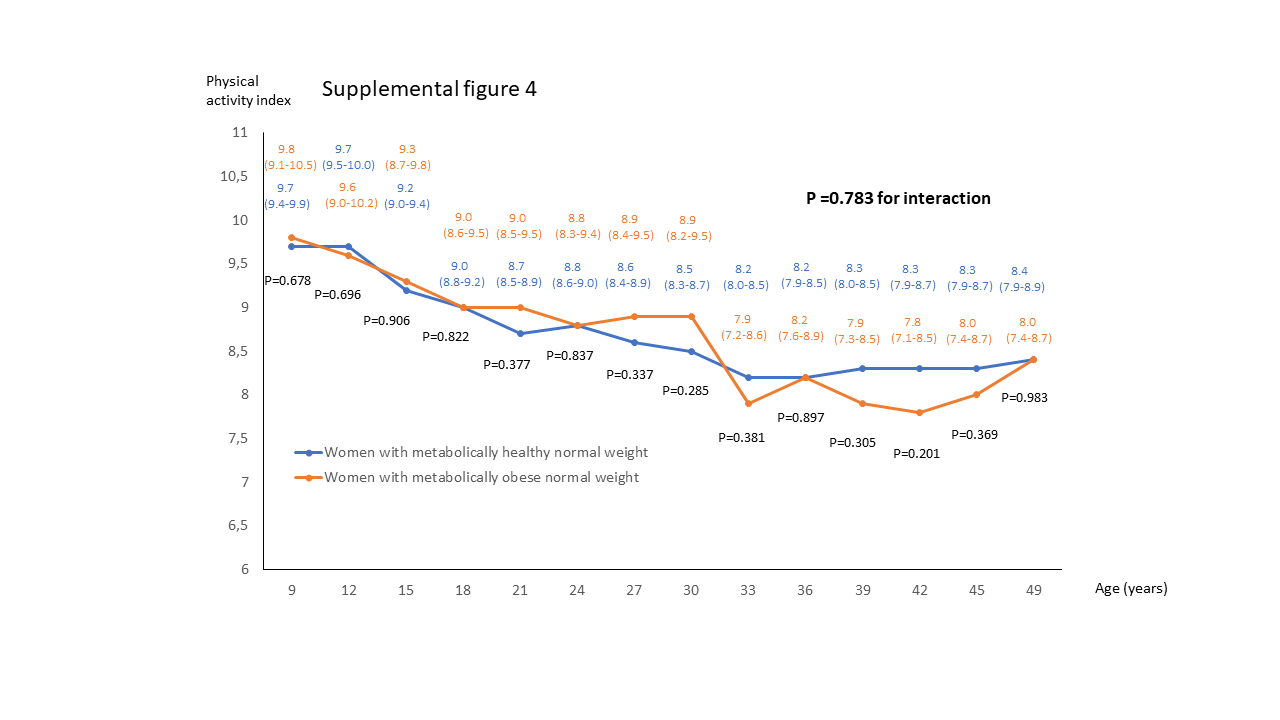

Supplement: Supplementary file 6 [file Image_6.tif]

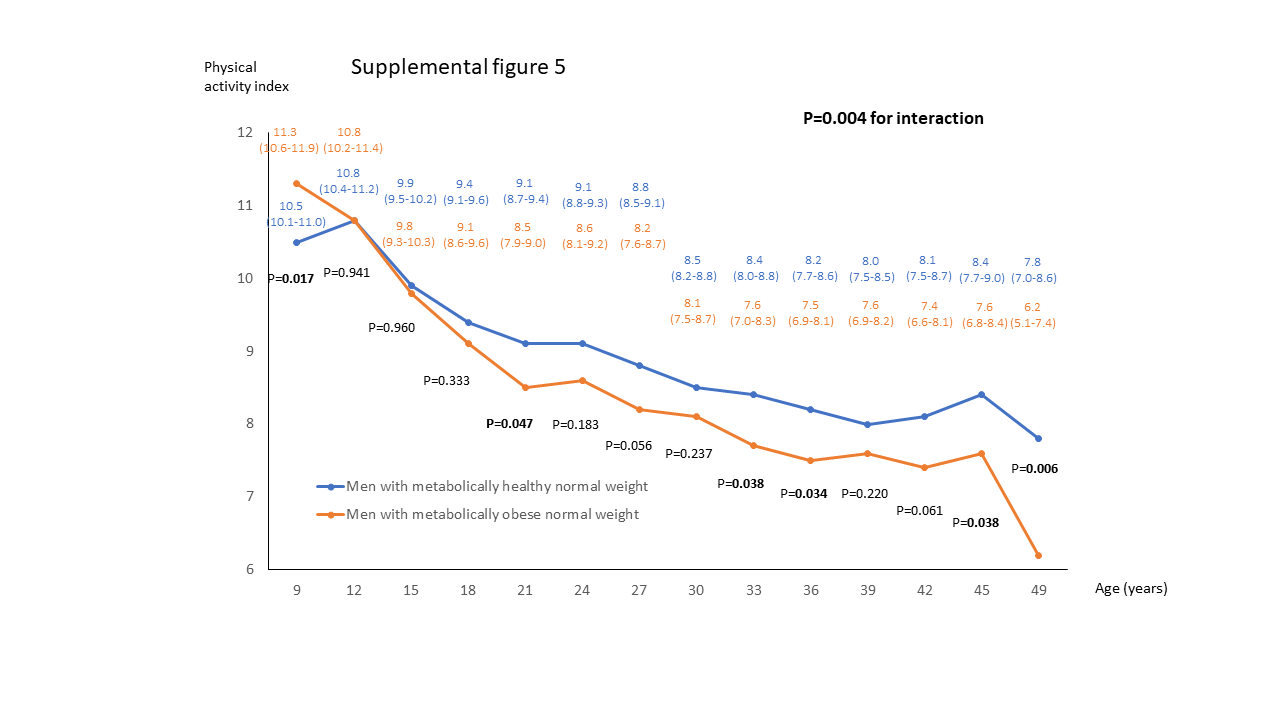

Supplement: Supplementary file 7 [file Image_7.tif]
